# Risk and protective factors to early childhood development during the COVID-19 pandemic

**DOI:** 10.1590/1980-220X-REEUSP-2022-0196en

**Published:** 2022-10-03

**Authors:** Priscila Costa, Evelyn Forni, Isabella Amato, Renata Longhi Sassaki

**Affiliations:** 1 Universidade Federal de São Paulo, Escola Paulista de Enfermagem, Departamento de Enfermagem Pediátrica, São Paulo, SP, Brazil.; 2 Centro de Estudos e Pesquisas “Dr. João Amorim”, São Paulo, SP, Brazil.; 3 Hospital e Maternidade São Luiz, São Paulo, SP, Brazil.; 4 Diversey, São Paulo, SP, Brazil.

**Keywords:** Child Development, Pediatric Nursing, Child Health, Primary Health Care, Desarrollo Infantil, Enfermería Pediátrica, Salud Infantil, Atención Primaria de Salud., Desenvolvimento infantil, Enfermagem Pediátrica, Saùde da Criança, Atenção Primária à Saùde

## Abstract

**Objective:**

To analyze the risk and protective factors to the development of children under three years of age during the COVID-19 pandemic.

**Method:**

Cross-sectional, quantitative study carried out in three early childhood education centers in the city of São Paulo, Brazil, in October 2020. The data were collected with an online questionnaire. Risk and protection factors were measured with the *Primeira Infância Para Adultos Saudáveis* (Early Childhood For Healthy Adults) instrument and the children's development status was measured using the Caregiver Reported Early Development Instruments – CREDI.

**Results:**

The study included 108 parents and guardians of children up to three years of age. Living with grandparents and participating in cash transfer programs were protective factors for child development. The family being headed by a woman posed a significant risk factor for child development.

**Conclusion:**

Intersectoral actions to support families headed by women and access to cash transfer programs are essential for promoting equity opportunities for the development in early childhood.

## RESUMO

**Objetivo:**

Analisar os fatores de risco e proteção para o desenvolvimento de crianças menores de três anos durante a pandemia por COVID-19.

**Método:**

Estudo transversal de abordagem quantitativa realizado em três centros de educação infantil na cidade de São Paulo em outubro de 2020. Os dados foram coletados por meio de questionário online. Os fatores de risco e proteção foram mensurados com o instrumento Primeira Infância Para Adultos Saudáveis e o status do desenvolvimento da criança foi aferido por meio do instrumento *Caregiver Reported Early Development Instruments* – CREDI.

**Resultados:**

Participaram do estudo 108 responsáveis por crianças de até três anos. Morar com os avós e participar de programas de transferência de renda foram fatores de proteção para o desenvolvimento infantil. A família ser chefiada por mulher representou um fator de risco significativo para o desenvolvimento da criança.

**Conclusão:**

Ações intersetoriais de apoio às famílias chefiadas por mulheres e acesso aos programas de transferência de renda são essenciais para a promoção da equidade de oportunidades para o desenvolvimento de crianças na primeiríssima infância.

## RESUMEN

**Objetivo:**

Analizar los factores de riesgo y protección al desarrollo de los niños menores de tres años durante la pandemia de COVID-19.

**Método:**

Estudio transversal y cuantitativo realizado en tres centros de educación infantil de la ciudad de São Paulo, Brasil, en octubre de 2020. Los datos fueron recolectados a través de un cuestionario en línea. Los factores de riesgo y protección se midieron con el instrumento Primeira Infância Para Adultos Saudáveis (Primera Infancia para Adultos Saludables) y el estado de desarrollo de los niños se midió utilizando el instrumento *Caregiver Reported Early Development Instruments* – CREDI.

**Resultados:**

El estudio incluyó a 108 responsables de niños de hasta tres años. Vivir con los abuelos y participar en programas de transferencias monetarias fueron factores protectores al desarrollo infantil. La familia encabezada por una mujer representa un factor de riesgo significativo al desarrollo de los niños.

**Conclusión:**

Las acciones intersectoriales de apoyo a las familias encabezadas por mujeres y el acceso a programas de transferencias monetarias son esenciales para promover equidad de oportunidades al desarrollo en la primera infancia.

## INTRODUCTION

The period between pregnancy and the first three years of life – early childhood – lays the foundations for health, well-being, learning, and productivity throughout life and is a phase of high susceptibility to environmental influence^(1)^. The greatest threats to development in early childhood involve extreme poverty, lack of public security, gender inequity, violence, environmental toxins, and mental health problems, which can affect both children and their caregivers^(1)^.

The COVID-19 pandemic changed the lives and care routines of many families with young children. The work environment was moved to the house; children stopped attending early childhood education schools and performed fewer activities outdoors and with other children^(2)^. Social distancing, which leads to confinement to the domestic context, has brought new and major challenges for families with young children, such as close interaction for long periods; no routine of attending schools, day care centers, welfare centers, and sports and leisure activities; remote working parents; rearranging the environment to accommodate work demands and playing; overload of domestic work; work instability, unemployment, and financial problems; lack of or irregular support from health services and social and community assistance for families, separation of relatives, among others^(3)^. Therefore, the pandemic may have affected the development and well-being of children under three years old.

A study conducted in China in 2020, when the pandemic started, revealed functional and behavioral difficulties in children, such as excessive dependence on parents, inattention, worry, sleep problems, lack of appetite, nightmares, discomfort, and agitation^(4)^. Another study, conducted with 617 preschool children in Brazil, showed that the pandemic had negative impacts on the children's cognitive and language development, in addition to increasing educational inequities for those with lower socioeconomic status^(5)^. For caregivers, the pandemic may have led to reduced wages, unemployment, food insecurity, difficulties in guaranteeing housing, and restricted access to health, education, and social assistance services^(6)^.

Given the relevance of this theme, evidence on the repercussions of the pandemic on the mental and emotional health of children and their families was provided. Systematic literature reviews^(7,8)^ have demonstrated increased depressive symptoms, anxiety, suicidal ideation, and delayed motor and language development in children and adolescents during the pandemic. Increased symptoms of depression, anxiety, and post-traumatic stress were reported in children's relatives. These data may be related to adverse experiences in early childhood and a high risk of toxic stress. The more adverse experiences, the higher the risk of developmental delays and health problems in adulthood, such as depression, chronic diseases, cognitive problems, and illicit drug abuse^(7)^.

Although evidence on the repercussions of the pandemic on children and their families has been provided, an empirical study on how family characteristics and their life context may influence the development of children aged zero to three years during the pandemic is necessary and poses a knowledge gap. This study hypothesizes that the characteristics of children's relatives, such as education, occupation, and life context, including income, as well as participation of the family in social programs, and children living with caregivers who are alcohol or drug users, may influence early childhood development during the pandemic, constituting risk or protective factors. Therefore, this study aims to answer the following research question: What are the risk and protective factors to early childhood development during the COVID-19 pandemic?

Understanding the risk and protection factors in child development during the COVID-19 pandemic can contribute to directing and prioritizing actions, programs, and public policies focusing on promoting early childhood development as well as mitigating the repercussions of the pandemic on child development. Given the relevance of this theme, the objective of this study is to analyze the risk and protection factors to the development of children under three years of age during the COVID-19 pandemic.

## METHOD

### Design of Study

This is a cross-sectional, quantitative study.

### Population

The population comprised relatives or guardians of children enrolled in the centers for early childhood education (CECE) participating in this research.

### Local

The study was conducted in three public CECE in the city of São Paulo, Brazil, which were located in regions of high social vulnerability of the Jabaquara neighborhood.

### Selection Criteria

The participant inclusion criterion was to be a father, mother, or guardian of a 0-to 35-month-old child regularly enrolled in the early childhood education center. The exclusion criterion was not being a WhatsApp user.

### Sample Definition

Convenience sampling was employed. Relatives of 450 children aged 0 to 35 months and regularly enrolled in the three participating CECE were invited to participate in this study. However, only 108 guardians agreed to answer the research questionnaire. Participation was higher (90% of the answers) in one of the day care centers, possibly due to extensionist actions having been performed there by this study's coordinator with families, children, and educators for four years before the pandemic. For the three participating CECE, after data collection, a report with infographics of the main results of the study was made available as feedback to families and daycare directors. The report contained a synthesis of the findings and orientation for family care and actions promoting the development of children under three years of age.

### Data Collection

The data were collected through an electronic form sent to relatives or guardians of children enrolled in the three early childhood education centers in October 2020. The relatives were invited to participate in the research through an invitation forwarded to family groups of each CECE via WhatsApp. The invitation to participate in the survey containing the link to the electronic form was sent three times, being available to relatives for a total of 30 days. The research team monitored daily the relatives' responses, observing that no new responses were submitted in the last four days of October and ending the data collection period.

The data collection form contained questions about the identification of children and their guardians/caregivers, such as the name of the day care center in which the child was enrolled, the child's age in months, the caregiver's age, color, gender, and relation with the child (mother, father, other). In addition, it included variables related to risk and protective factors to child development and issues related to the status of child development.

Risk and protective factors to child development were verified using items from the “protection and safety” domain of the instrument Early Childhood for Healthy Adults (*Primeira Infância Para Adultos Saudáveis* – **PIPAS**)^(9)^, which is the first instrument for assessing the development of children from zero to five years old elaborated and validated in Brazil and capable of large-scale application with good reliability. The variables of this instrument included alcohol use during pregnancy (yes or no), smoking during pregnancy (yes or no), whether there was maternity leave (yes or no), family participation in a social or cash transfer program, such as *Bolsa Família* (yes or no), who lives with the child (father, mother, siblings, grandparents, partner, nanny, others), who is the head of the family or responsible for most of the house's income (mother, father, other), mother's age and education, head of household's occupation (employed or unemployed), monthly family income (<1 minimum wage, 1 to 3 wages, 4 to 5 wages, more than 5 wages), skin color of the child (white, black, brown, Asian, indigenous), child gender (female or male), whether maternal depression was diagnosed by a health professional (yes or no), and if the child lives with drug or alcohol users.

The outcome under study was child development status, measured with the short version of the Caregiver Reported Early Development Instrument – CREDI^(10,11)^, a population-level measure developed internationally to assess the overall development of children aged 0–35 months in the motor, language, cognition, socio-emotional and mental health domains. This instrument contains 20 specific questions for each age group (0–5, 6–11, 12–17, 18–23, 24–29, and 30–35 months old), which are directed to the child's primary caregiver, using the yes/no answer scale^(11)^. This is a valid, reliable, and acceptable early childhood development measure for children under three years of age in Brazil and has been previously used^(10)^ for data collection from electronic forms. The CREDI short form provides a score representing child development status. This version does not present specific scores for the different development domains^(11)^.

### Data Analysis and Treatment

The data were stored in the Excel software and analyzed with the SPSS software. Categorical and numerical variables are presented through descriptive statistics. To find associations between explanatory variables and continuous outcome, the linear regression model was used. Linear regression enabled analyzing the relationship between risk and protective factors to child development and child development status, a continuous variable which is the outcome of this study.

### Ethical Aspects

The study was carried out in accordance with national and international ethical standards of research involving human beings. The research project was submitted to the Research Ethics Committee of a public university in São Paulo, Brazil (opinion number 4.068961). The relatives were informed of the study's objectives and provided with the Informed Consent Form (ICF), according to ethical guidelines for research in virtual environments.

## RESULTS

The study included 108 guardians of children under three years of age. Most were the child's mother (94.5%), followed by father (2.75%), and grandparents (2.75%). Regarding caregiver age, the mean maternal age was 30 years, with a minimum of 17 years and a maximum of 46 years. Regarding paternal age, the mean age was 33 years, with a minimum of 19 years and a maximum of 57 years. Regarding the children's characteristics, most were white (50.5%), male (52.3%), and lived with their mother (96.3%) and father (66.1%). The other sociodemographic characteristics are presented in Table 1.

**Table 1:** Sociodemographic characteristics of the children's families – São Paulo, SP, Brazil, 2020.

Sociodemographic characteristics	N = 108 (100%)
Family type*	
Nuclear family	73 (67.6%)
Single parent	35 (32.4%)
**Family headed by woman**	
Yes	56 (51.8%)
No	51 (47.2%)
**Maternal occupation**	
Unemployed	62 (57.4%)
Employed	46 (42.6%)
**Maternal education**	
Primary Education	18 (16.7%)
Secondary Education	68 (63.0%)
Higher Education	22 (20.3%)
**Maternity leave**	
Yes	59 (54.6%)
No	49 (45.4%)
**Smoked during pregnancy**	
Yes	12 (11.1%)
No	96 (88.9%)
Alcohol use during pregnancy*	
Yes	6 (5.6%)
No	101 (94.4%)
Maternal depression*	
Yes	11 (10.3%)
No	96 (89.7%)
**Child lives with relative who drinks alcohol**	
Yes	6 (5.6%)
No	102 (94.4%)
**Child lives with relative who uses drugs**	
Yes	1 (0.9%)
No	107 (99.1%)
Monthly family income*	
Less than 1 minimum wage	40 (46.5%)
1 to 3 minimum wages	37 (43%)
3 to 5 minimum wages	9 (10.5%)
**Participates in cash transfer program**	
Yes	42 (38.9%)
No	66 (61.1%)

* Data could not be collected from all families.

Table 2 presents data on the status of child development, measured using the short form of the Caregiver Reported Early Development Instrument – **CREDI**, which provides a score for the child's overall development.

**Table 2:** CREDI score according to child age group – São Paulo, SP, Brazil, 2020.

Child age group	Mean	Standard deviation	Median	Maximum	Minimum
Under 6 months	46.0	0.86	44.3	46.9	21.8
6 to 11 months	46.6	0	46.6	49.9	29.1
12 to 17 months	50.2	2.25	49.7	56.9	38.3
18 to 23 months	53.3	3.52	52	60.5	46.2
24 to 29 months	57.4	2.68	57.4	61.9	48.3
30 to 35 months	58.2	2.46	57.7	62.4	48.7
Total sample (n = 108)	51.9	1.9	51.2	56.4	38.7

The sociodemographic characteristics were analyzed with linear regression for their association with child development status. The objective of this analysis was to identify which ones represented risk factors or protective factors to child development. The data in Table 3 revealed that the family's participation in a cash transfer program and children living with grandparents are statistically significant factors of protection to child development. The identified risk factor was the family being headed by a woman.

**Table 3:** Risk and protective factors to child development – São Paulo, SP, Brazil, 2020.

Risk and protective factors to child development	Coefficient	P-value
**Protective factors**		
Employed mother	0.88	0.06
Mother with higher education	4.68	0.62
Mother with secondary education	4.61	0.37
Maternity leave	1.49	0.94
Monthly family income from 1 to 3 salaries	1.03	0.85
Monthly family income from 3 to 5 salaries	1.67	0.92
Family participates in cash transfer program	2.63	0.005
Child living with grandparents	3.17	0.003
**Risk factor**		
Single parent	0.94	0.62
Mother with primary education	4.71	0.39
Child lives with relative who uses drugs	4.63	0.49
Maternal depression	1.47	0.3
Alcohol use during pregnancy	1.93	0.48
Smoked during pregnancy	1.91	0.42
Family headed by woman	−1.55	0.071

## DISCUSSION

The findings of this study revealed that family participation in cash transfer programs and child living with grandparents were protective factors to the development of children under three years of age during the **COVID-19** pandemic. On the other hand, families headed by women were a risk condition to child development. These results emphasize the importance of actions, programs, and public policies aimed at families with young children living in poverty-related situations of vulnerability, with scarce support network, and in which women are responsible for most of the family's income^(1)^.

Poverty directly impacts the development of children, as it reduces opportunities for their full development. Our findings show that family participation in a cash transfer program was a significant protective factor to integral child development. There is evidence that cash transfer programs increase the family's food security, leading to improvements in food quality and the number of meals, as well as child well-being^(12)^. Corroborating this study's findings, a study^(13)^ verifying the effects of cash transfer programs on integral child development identified reduced socio-emotional problems in children, improved cognitive development, and greater access to electricity, gas stoves, and food. The cash transfer policy plays an important role not only for the individual, but also for society, including the association between child poverty and a greater likelihood of young people developing externalizing disorders, such as attention deficit and hyperactivity disorder, especially among women^(14)^.

Living with grandparents was presented as a protective factor to child development. Regarding childcare, a study^(2)^ conducted with 1,036 Brazilian families during the pandemic revealed that grandparents played an important role in early childhood care. During the pandemic, grandparents, as well as parents, recognized the importance of playing and storytelling as interactions that favor child development^(2)^.

However, such study^(2)^ revealed that 34% of mothers reported overload and 36% reported exhaustion during the pandemic. Recognizing that the pandemic has affected women and men in different ways is a fundamental step to understand the primary and secondary effects of health emergencies in different individuals and communities, as well as for interventions and public policies that promote gender equity to be formulated and implemented^(15)^.

The pandemic impacts women mostly, among several reasons, due to their greater vulnerability in the labor market, in addition to their social role as responsible for most domestic work and child care, which increased substantially during the pandemic^(16)^. Public policies and actions aimed at gender equity are mandatory to cope with the burden of female heads of family. Thus, cash transfer programs are more beneficial for women than men, especially in times of increased unemployment and poverty^(16)^. During the pandemic, measures to support the needs of families and work-life balance in high-income countries, such as the expansion of parental leave or the inclusion of benefits to support child care, were important when schools were closed^(16)^. However, such actions or policies have not been implemented to support families in low- and middle-income countries, such as Brazil.

The pandemic is a major stressful event with no precedents for children, families, communities, cities, and even countries^(3)^. However, it must be understood that **COVID-19** is not the only epidemic to threaten humanity and will not be the last^(3)^; therefore, learning from this experience can be useful to direct actions, programs, and public policies, especially those for vulnerable families whose developmental niches presented a high level of adversity from the start. Supporting families for the maintenance of the foundations of competence, relationship, and autonomy of individuals is fundamental for adaptive coping in this adverse situation^(3)^.

Although this study found that the participation of families in cash transfer programs, living with grandparents, and female heads of family influence the development of children in the first three years of life during the **COVID-19** pandemic, its limitations include its cross-sectional design and a limited sample size. There is evidence that maternal education^(17)^, maternal depression^(18)^, and the use of tobacco, alcohol, or other drugs^(19)^ during pregnancy negatively influence child development. However, probably due to the study design and sample size limitations, these associations were not statistically significant in this study. Therefore, further research with population samples and prospective and longitudinal data collection, such as intergenerational cohort studies, are essential to understand the factors that influence human development over time and assess the real impacts of the pandemic.

Implications of this study for nursing, especially for the field of primary health care, include the importance of care that fosters integral child development in expanded childcare clinics^(20)^. Nurses have the important role of promoting and protecting children's health by providing care beyond physical development while considering the mental health of children and their caregivers, as well as their needs and life context.

Given the complexity of children's and their families' needs, actions aimed at intersectoral integration are fundamental for the promotion of development in early childhood. This means promoting integration between different levels of Health, Education, Social Development, Justice, and other systems^(20)^. Problemsolving tends to become more effective when the various sectors jointly define priorities for the development of the local child population and interfaces are established, articulating social policies and the initiatives implemented in the municipality^(20)^.

Nurses play an important role in qualifying public policies, programs, and actions to support families with children under three years of age. Essential measures include providing support for the most vulnerable families to have access to cash transfer programs, such as Bolsa Família and Auxílio Brasil, as well as strengthening the parental skills of child caregivers, such as grandparents. In addition, nurses can strengthen families headed by women and, consequently, the child's care network, supporting their access to quality public early childhood education.

The promotion of equity of opportunities in early childhood and the reduction of the repercussions of the pandemic on child development involve intersectoral actions, programs, and public policies aimed at reducing poverty, strengthening parental care, and supporting gender equity, especially for socially vulnerable families.

## CONCLUSION

The results of this study emphasized the protective role of living with grandparents for child development, as well as cash transfer programs and the need to prioritize support for female heads of family to promote the development of children under three years of age.

Promoting early childhood development must be a priority, given the importance of this stage of life for human development and for society. Actions to strengthen the skills of adult caregivers to promote child development, including grandparents, as well as intersectoral actions to support families headed by women and the guarantee of access to cash transfer programs and early childhood education, are essential to promote equity opportunities for early childhood development.
